# Tuberculosis reactivation in hematopoietic stem cell transplant recipients: a preemptive strategy in an endemic country

**DOI:** 10.1016/j.jctube.2025.100565

**Published:** 2025-09-27

**Authors:** Irma Karen Pellón-Téllez, Omar Eduardo Fernandez-Vargas, Patricia Cornejo-Juárez, Alexandra Martin-Onraet, Luis Felipe Rubalcava-Lara, Rosa Adriana Alvidrez-González, Andrés Bonilla-Salcedo, Luis Valero-Saldaña, Brenda Lizeth Acosta-Maldonado

**Affiliations:** a Department of Hematology, Instituto Nacional de Cancerología, Mexico, Mexico; b Department of Infectious Diseases, Instituto Nacional de Cancerología, Mexico, Mexico

**Keywords:** Latent Tuberculosis, Hematopoietic stem cell transplant, Tuberculosis prophylaxis, Tuberculosis reactivation

## Abstract

**Background:**

Tuberculosis (TB) remains a global public health issue, particularly in developing countries. Hematopoietic stem cell transplant (HSCT) recipients are at increased risk for TB due to immunosuppression.

**Methods:**

This retrospective study (2005–2022) assessed a preemptive latent tuberculosis infection (LTBI) screening and treatment strategy in 338 HSCT recipients and their donors. Screening included tuberculin skin test (TST) or QuantiFERON-TB Gold (QFT), and pulmonary imaging, with positive cases receiving isoniazid (INH) therapy before HSCT. Statistical analyses compared TB reactivation rates, overall survival (OS), and relapse-free survival (RFS) across HSCT groups.

**Results:**

Of 338 patients, screening involved TST in 325 and QFT in 13 cases, resulting in 94 (27.8%) patients with LTBI. INH therapy was completed by 83 patients, with 5 discontinuing due to hepatotoxicity. The cumulative incidence of active TB was 0.6%, and one patient who received INH prophylaxis developed LTBI reactivation. LTBI status did not affect OS or RFS.

**Conclusions:**

Our study demonstrates that the use of LTBI protocols incorporating TST/QFT and chest CT-scans, followed by INH in positive cases, was safe and is an accessible option among HSCT recipients in high-burden regions.

## Highlights

• LTBI remains a concern for cell therapy recipients, especially in endemic areas.
• We assessed a preemptive screening strategy in 338 HSCT recipients, 27.8% had LTBI.
• INH for LTBI was well tolerated; and only one (1.2%) patient had TB reactivation.
• Latent tuberculosis had no impact on overall survival or relapse rates.

## 1 Introduction

Tuberculosis (TB), an infectious disease primarily affecting the lungs, remains a significant public health concern, particularly in developing countries, despite being both preventable and curable. According to the World Health Organization (WHO), an estimated 10.6 million people worldwide contracted TB in 2021, resulting in 1.6 million deaths [1]. High-burden countries (HBCs), as defined by the WHO (≥20 cases per 100,000), include nations in Africa, Latin America, and Asia, where new TB cases continue to increase [1]. Mexico is considered an endemic country for latent TB infection (LTBI), with an incidence rate of 28 cases per 100,000 population annually, as reported by the World Bank Group [2].

TB infection is recognized as a spectrum, reflecting a range of states influenced by bacterial load, host immune response, environmental factors, and genetic predisposition [3]. At one end of the spectrum is LTBI, where the bacteria is present but inactive, causing no clinical symptoms. Intermediate states may exist where the bacteria begin to replicate, yet the host’s immune system still contains the infection, leading to subclinical or incipient TB. At the other end is active TB infection (ATBI), characterized by symptomatic disease, which can involve multiple organs and may arise from primary infection or reactivation of latent TB [3,4].

TB infection is recognized as a spectrum, reflecting a range of states influenced by bacterial load, host immune response, environmental factors, and genetic predisposition [3]. At one end of the spectrum is LTBI, where the bacteria is present but inactive, causing no clinical symptoms. Intermediate states may exist where the bacteria begin to replicate, yet the host’s immune system still contains the infection, leading to subclinical or incipient TB. At the other end is active TB infection (ATBI), characterized by symptomatic disease, which can involve multiple organs and may arise from primary infection or reactivation of latent TB [3,4].

Vulnerable populations for TB infection include individuals with comorbidities such as diabetes mellitus, silicosis, end-stage renal disease, malnutrition, recent TB infection, patients receiving glucocorticoids or biological agents, and those in immunocompromised states, including persons living with HIV and recipients of hematopoietic stem cell transplants (HSCT) [5].

The cumulative lifetime risk of LTBI reactivation to active disease is estimated to be around 5–15 %, however, this risk can increase from 10 to 40 times in HSCT recipients compared to healthy controls [3]. Pulmonary infection is the primary site of TB in these patients, with a mortality rate ranging from 0 to 50 % [6]. According to the European Conference on Infections in Leukaemia (ECIL), the rate of ATBI in HSCT recipients is estimated to be 2.7 % (ranging from 1.5 % to 16.0 %) in regions with high TB incidence (≥100 cases per 100,000 population); 2.2 % (ranging from 0.2 % to 8.5 %) in areas with intermediate TB incidence; and 0.7 % (ranging from 0.4 % to 2.3 %) in low incidence regions (<20 cases per 100,000 population [7].

The WHO guidelines for TB preventive therapy (TPT) outline treatment strategies for groups at the highest risk of developing active TB. TPT is considered in migrants from countries with high TB burden, people with silicosis, health workers, homeless persons, prisoners, and illicit-drug users, and patients receiving immunosuppressive therapy or undergoing either solid organ transplantation or HSCT [4]. However, the efficacy of TPT is variable, particularly in the context of HSCT, with only a few studies demonstrating a benefit with isoniazid (INH) therapy [8].

Although current IDSA and ECIL guidelines recommend screening for LTBI in HSCT recipients [7,9], the implementation of routine protocols remains inconsistent across transplant centers. According to ECIL data, only an estimated 36 % of HSCT centers have adopted a formal LTBI screening strategy [7]. We conducted a literature review to identify current strategies and their efficacy in cell therapy recipients. We explored the effectiveness of a preemptive strategy in a tertiary referral oncology center consisting of either a tuberculin skin test (TST) or QuantiFERON-TB Gold (QFT), along with a pulmonary CT scan, followed by INH therapy for positive cases, before HSCT.

## 2 Materials and methods

We conducted a retrospective study of patients aged 18 years or older at the time of HSCT, who underwent either allogeneic HSCT (allo-HSCT) or autologous HSCT (auto-HSCT) and their donors, at a tertiary referral oncology center, between January 2005 and December 2022. The main objective was to describe a preemptive strategy of LTBI screening and INH therapy for positive cases, and the rates of TB reactivation to ATBI among HSCT recipients.

We collected clinical, microbiological, and outcome data from electronic medical records, including baseline disease characteristics, type of transplant, TB screening strategy, INH therapy and adherence, and HSCT outcomes. Patients were categorized into auto-HSCT or allo-HSCT groups based on the type of HSCT they received. Individuals with incomplete medical records, those who did not complete the preemptive screening strategy, or those with less than 6 months of follow-up after HSCT were excluded from the analysis.

The preemptive LTBI screening strategy consisted of either TST or QFT, combined with a chest X-ray. According to institutional protocol, all patients with a positive TST or QFT underwent chest CT (within three months) to exclude ATBI. Many had already undergone CT or PET-CT imaging as part of their hematologic workup to assess remission status. For those without recent imaging and positive TST/QFT results, a chest CT was performed to complete the evaluation. Further testing in positive patients such as *M. tuberculosis* cultures or nucleic acid amplification testing (NAAT), including GeneXpert were performed at the treating physician's discretion.

Regarding TST and QFT testing rationale, TST detects LTBI by intradermally injecting a small amount of purified protein derivative (PPD) of *Mycobacterium tuberculosis* (Mtb). After 48 to 72 h, a hypersensitivity reaction with an induration of ≥ 5 mm was considered positive, as per ATS/IDSA/CDC guidelines [10], ECIL guidelines [7], and CDC recommendations [11]. If the initial TST was negative, patients proceeded to a two-step testing (second TST) at least two weeks later, to account for a booster effect. Donors were considered positive with a TST of ≥ 10 mm, following the cut-off point recommended for non-immunosuppressed patients [11]. QFT, a serologic interferon-gamma release assay (IGRA), measures the immune response to Mtb antigens [9]. In the event of an indeterminate result, patients were asked to undergo a TST; however, no indeterminate QFT results were observed.

After evaluation, patients diagnosed with ATBI received appropriate Mtb treatment, while those with LTBI began INH therapy at a daily dose of 300 mg for nine months. Per protocol, it was advised that patients receive at least two months of INH prior to HSCT, however, transplantation was not delayed if this was not achieved. The goal was to complete the full nine-month course.

**Fig. 1:**
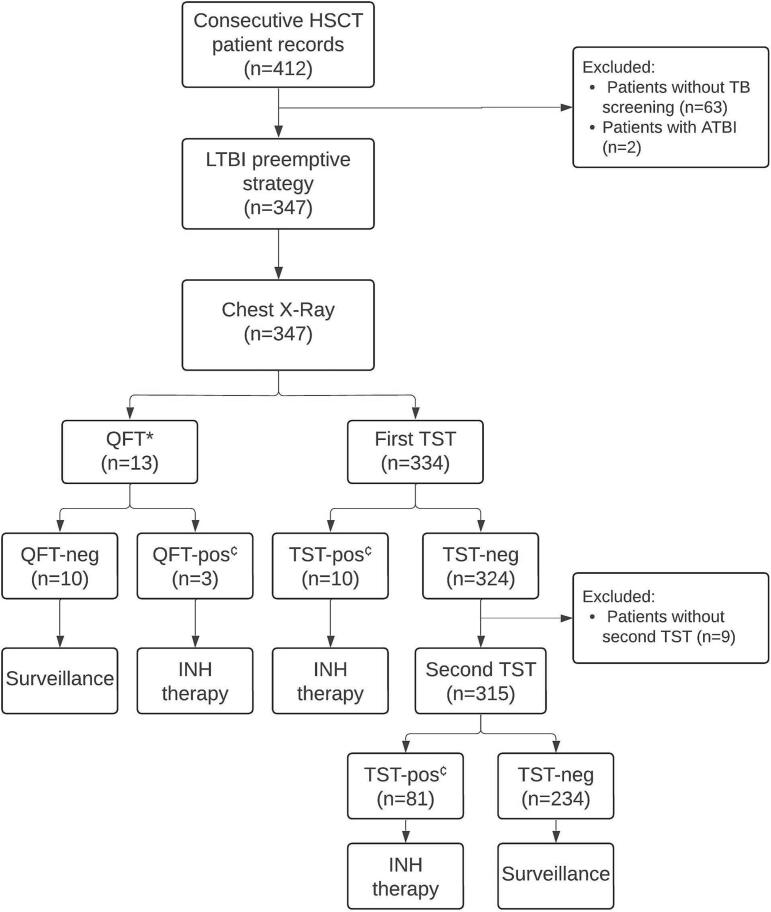
Flow diagram of HSCT recipients included in the study. *If QFT was performed, only one TB screening test was needed. ^¢^Patients with a positive TST or QFT result were referred for chest CT imaging to evaluate for active tuberculosis. ATBI, active tuberculosis infection; INH, isoniazid; HSCT, hematopoietic stem cell transplant; LTBI, latent tuberculosis infection; QFT, QuantiFERON-TB Gold Plus; TST, Tuberculin Skin Test.

TB reactivation was defined as the development of ATBI in individuals previously diagnosed with LTBI during follow-up [8]. The preemptive strategy above is outlined in Fig. 1.

Categorical variables are described using frequencies and percentages, while quantitative data are presented using medians and ranges. Statistical analysis involved comparing categorical variables using Fisher’s exact test and employing unpaired t-tests for normally distributed continuous variables. In contrast, the Mann-Whitney test was used for non-normally distributed continuous variables. The cumulative incidence of TB was calculated as the number of new active TB cases during the follow-up period, divided by the total number of patients included in the preemptive strategy. The TB reactivation rate was defined as the proportion of new cases of ATBI in patients diagnosed with LTBI, and compared between HSCT groups. Overall survival (OS) and relapse-free survival (RFS) were analyzed using the method described by Kaplan and Meier. All data were processed using GraphPad Prism (version 9.5.2) and RStudio (version 2023.12.1 + 402), with a statistical significance p-value set at less than 0.05.

Approval for the study was obtained from the Institutional Ethics Committee, and the study was conducted in accordance with the Declaration of Helsinki.

## 3 Results

### 3.1 Patient characteristics

Of the 412 medical records reviewed, 338 (82.0 %) patients met the inclusion criteria. Among the 74 patients excluded, 63 had not undergone tuberculosis screening tests, nine had incomplete screening protocols, and two had a history of ATBI prior to HSCT. The median age at disease diagnosis was 36 years (range, 12–67), and the median age at HSCT was 40 years (range, 18–69). The cohort comprised 195 (57.7 %) males and 143 (42.3 %) females, all identified as Mexican mestizos (Table 1).

**Table 1:** Baseline demographics of 338 HSCT recipients undergoing a preemptive TB strategy.

Parameters	auto-HSCT, n = 210	allo-HSCT, n = 128
Sex, n (%) Female Male	82 (39.0 %) 128 (61.0 %)	61 (47.7 %) 67 (52.3 %)
Age at diagnosis, median (range)	36 (17–67)	36 (12–60)
Age at HSCT, median (range)	40 (18–69)	40 (18–61)
Diagnosis, n (%)	NHL, 86 (40.9 %) MM, 73 (34.8 %) HL, 45 (21.4 %) Solid tumor, 5 (2.4 %) WM, 1 (0.5 %)	ALL, 51 (39.8 %) AML, 36 (28.2 %) CML, 15 (11.8 %) HL, 9 (7.0 %) MDS, 5 (3.9 %) NHL, 4 (3.1 %) AA, 4 (3.1 %) DCN, 3 (2.3 %) PMF, 1 (0.8 %)
Previous treatment lines, median (range)	2 (1–3)	2 (1–4)
Conditioning regimen, n (%)	PEAM*, 126 (60.0 %) MEL200, 29 (13.8 %) BuMEL, 23 (10.9 %) BorMEL, 21 (10.0 %) Other, 11 (5.3 %)	BuCy, 73 (57.0 %) FluBu, 21 (16.4 %) FluBuCy, 30 (23.4 %) FluCy + ATG, 4 (3.2 %)
LTBI, n (%) Recipient Donor	54 (25.7 %) N.A.	40 (31.3 %) 19 (14.8 %)
Number of deaths, n(%)	36 (17.1 %)	57 (44.5 %)
Cause of death, n (%) Relapse Infection GvHD Hepatic failure Veno-occlusive disease Graft failure Hemorrhage Unknown	22 (61.2 %) 11 (30.7 %) N.A. 1 (2.7 %) 0 0 0 2 (5.4 %)	28 (49.1 %) 19 (33.3 %) 7 (12.2 %) 0 1 (1.8 %) 1 (1.8 %) 1 (1.8 %) 0
*See reference [32]. AA, Aplastic Anemia; ALL, Acute Lymphoblastic Leukemia; AML, Acute Myeloid Leukemia; CML, Chronic Myeloid Leukemia; DCN, Dendritic Cell Neoplasms; GvHD, Graft versus Host Disease; HL, Hodgkin lymphoma; HSCT, Hematopoietic Stem Cell Transplant; LTBI, Latent Tuberculosis Infection; MDS, Myelodysplastic Neoplasm; MM, Multiple Myeloma; N.A., non available; NHL, non-Hodgkin lymphoma; PMF, Primary Myelofibrosis; TST, Tuberculin Skin Test; WM, Waldenström’s Macroglobulinemia.		

By diagnosis, the distribution of patients was as follows: 53 with acute lymphoblastic leukemia (ALL), 36 with acute myeloid leukemia (AML), 15 with chronic myeloid leukemia (CML), 88 with non-Hodgkin lymphoma (NHL), 54 with Hodgkin lymphoma (HL), 73 with multiple myeloma (MM), 5 with myelodysplastic syndromes (MDS), 5 with solid neoplasms, 4 with aplastic anemia (AA), 3 with dendritic-cell neoplasms, 1 with myelofibrosis, and 1 with Waldenström macroglobulinemia (WM).

The median lines of therapy before undergoing HSCT was 2 (range 1–4). Overall, 210 (50.9 %) underwent auto-HSCT, and 128 (31.1 %) underwent allo-HSCT. Within the allogeneic group, 99 (77.3 %) received a matched sibling donor (MSD) transplant, and 29 (22.7 %) had an haploidentical transplant. Furthermore, allo-HSCT recipients received myeloablative conditioning in 106 (82.8 %), and 22 (17.2 %) received reduced-intensity conditioning (RIC).

### 3.2 Latent tuberculosis screening protocol

The LTBI protocol for patients consisted of TST in 325 (96.2 %) patients and QFT in 13 (3.8 %) patients. The initial TST was positive in only 10 transplant recipients, necessitating retesting for the remaining 315 patients. Of these, 81 patients were positive after the booster dose. Only three patients with a QFT were diagnosed with LTBI. HSCT donors all had TST and chest imaging.

Overall, LTBI was diagnosed in 94 (27.8 %) HSCT recipients, with 24 showing abnormal chest imaging results and 9 having a history of community exposure to Mtb. Abnormal chest imaging findings included six patients with multiple pulmonary nodules and one patient with granuloma suggestive of fungal infections, four patients with calcified granulomas, three with calcified nodules, and three with nodules classified as secondary to HL, including one patient with progressive disease. Additionally, two patients presented with pulmonary fibrosis, 2 with nodules due to systemic NHL involvement, and one patient each with ground-glass opacities due to COVID-19, nodules associated with *Staphylococcus aureus* pneumonia, and nodules due to histoplasmosis.

Among the 128 allo-HSCT donors, 19 (14.8 %) tested positive for LTBI, with only one showing abnormal chest imaging, presenting with calcified nodules due to a prior history of TB. Additionally, eight allo-HSCT pairs had both donor and recipient testing positive for LTBI.

Exploring LTBI-positive and negative HSCT recipients, no significant differences were found in age at transplant (41.4 vs. 40.1 years, respectively; p = 0.430) or in the type of transplant, with 25.7 % LTBI positivity in the auto-HSCT group compared to 31.3 % in the allo-HSCT group (p = 0.315). However, there was a significant female predominance among LTBI-positive cases (54.4 % vs. 37.8 %, p = 0.0067).

A total of 88 (93.6 %) HSCT recipients with LTBI initiated INH therapy. Of these, 83 patients completed the 9-month INH regimen, while 5 (5.7 %) discontinued therapy due to hepatotoxicity. Among the six patients who did not receive LTBI treatment, no cases of TB reactivation were reported in 4 patients, while the other two patients were lost to follow-up.

### 3.3 ATBI and TB reactivation

Two cases of ATBI were documented. The first case involved an auto-HSCT recipient with NHL and type 2 diabetes, who had a TST of 15 mm before HSCT. Without TB symptomatic disease, LTBI was diagnosed, and the patient had been on INH for four months. During the transplant nadir, she developed a cough and pulmonary infiltrates, visible on chest radiography. A Ziehl-Neelsen (ZN) stain on bacilloscopy tested positive for TB, and she subsequently completed a six-month course of antituberculous treatment.

The second case consisted of an auto-HSCT recipient who was HIV-positive and developed tuberculous lymphadenitis two months post-transplant. This patient had a negative TST before HSCT and a CD4 count of less than 200 cells/mm^3^. He presented with cervical lymphadenopathy, and the diagnosis was confirmed through histopathology, which revealed granulomatous lymphadenitis. However, TB NAAT, ZN staining, and cultures were all negative. He received a seven-month course of anti-TB treatment, leading to clinical improvement.

Overall, the cumulative incidence of ATBI was 0.6 % (2/338). Among patients with LTBI who received INH therapy, only one case of TB reactivation was reported, for a reactivation rate of 1.2 %.

### 3.4 Overall and relapse-free survival

The median follow-up was calculated using the reverse Kaplan-Meier method, for 52 months (range 1–164), with 93 deaths registered, resulting in a mortality rate of 27.5 %. The primary causes of death included relapse, the most prevalent at 50 (53.8 %) cases, followed by infection in 30 (32.3 %) patients. Additionally, graft-versus-host disease (GvHD) accounted for 7 (7.5 %) cases, while veno-occlusive disease, graft failure, hepatic failure, and hemorrhage accounted for one case each. There were 2 patients for whom the cause of death was not reported.

**Fig. 2:**
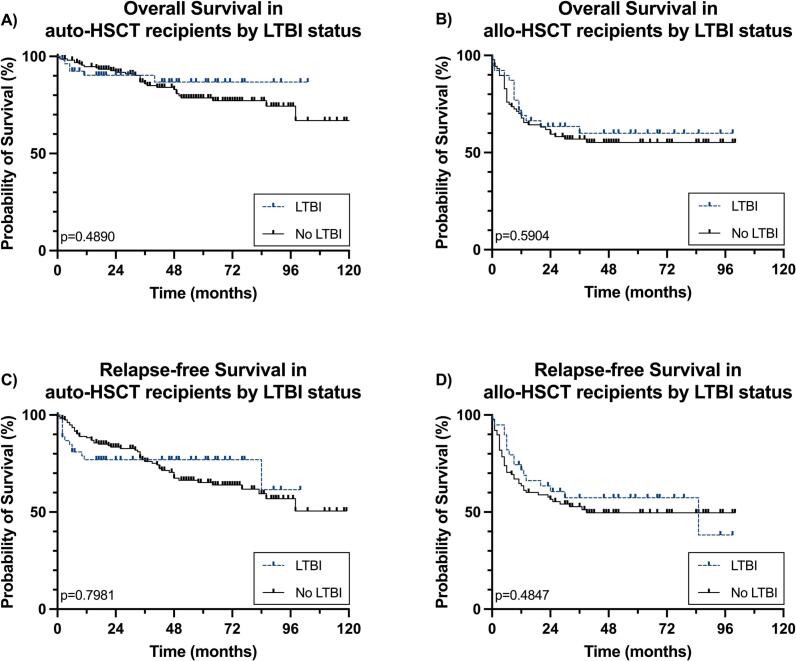
Kaplan-Meier curves for overall survival (OS) and relapse-free survival (RFS) in HSCT recipients based on LTBI-status and type of transplant. Panel A) illustrates the OS for auto-HSCT recipients, while panel B) depicts OS for allo-HSCT recipients. Panel C) presents RFS in auto-HSCT recipients, and panel D) shows RFS for allo-HSCT recipients.

OS was not reached for the entire cohort or when stratified by transplant type. The 24-month OS rates were 91.4 % for patients who underwent auto-HSCT and 62.6 % for those who received allo-HSCT, with this difference reaching statistical significance (p < 0.0001). LTBI was not associated with a worse OS in the overall cohort (HR 1.124, 95 %CI 0.688–1.836, p = 0.640), nor when stratified by transplant type. This held for both auto-HSCT (Fig. 2A) and allo-HSCT recipients (Fig. 2B).

A total of 89 relapses were recorded, with a median post-transplant RFS not yet reached. The 24-month RFS was 79.3 % overall, 83.7 % for auto-HSCT recipients, and 71.9 % for the allo-HSCT group, indicating a significant difference between the two types of transplants (p = 0.0003). LTBI was not associated with poorer RFS (HR 1.076, 95 % CI 0.713–1.625; p = 0.726). A total of 22/94 (23.4 %) patients in the LTBI group relapsed, compared to 67/244 (27.5 %) in the non-LTBI group. Moreover, RFS was not inferior in LTBI-positive cases compared to LTBI-negative cases when analyzed by type of transplant, both for auto-HSCT (p = 0.7981) (Fig. 2C) and allo-HSCT (p = 0.4847) (Fig. 2D).

## 4 Discussion

Our analysis identified a total of 40 (31.3 %) allo-HSCT recipients, 54 (25.7 %) auto-HSCT recipients, and 19 (14.8 %) donors with LTBI; thus, the highest prevalence was among allo-HSCT recipients. This increased prevalence, despite the smaller number of allo-HSCT procedures, could be attributed to underlying hematologic diagnosis, such as acute leukemia, and the patients’ baseline immune status. The prevalence of LTBI in healthy donors often reflects that of the general population. In Mexico, the prevalence of LTBI is highly variable, with reports reaching as high as 50 % in certain groups and regions. The donor prevalence in our study (roughly 15 %) likely represents a mixed population from both intermediate and high-burden areas across the country.

Despite being a region with areas highly endemic for TB, data on HSCT recipients in Latin America (LATAM) remain scarce. In our cohort, we observed a prevalence of LTBI of 27.8 %, which is similar to other reports from the region. In Mexico, Bourlon et al. documented a prevalence of LTBI of 20 % using TST screening [12], while in Brazil, LTBI prevalence ranged from 8.7 % to 17.1 %, using both TST and QFT testing (Table 2) [13,14]. No additional reports from other LATAM countries were identified, indicating a significant gap in the literature. Our findings contribute to the limited information available on this topic in LATAM, a region where the burden of TB remains high.

**Table 2:** Reported studies and strategies of LTBI and ATBI in HSCT, immune effector cell and T-cell directed therapy recipients.

Study	Year	Country	High Burden Country#	Therapy	LTBI prevalence in the study (%)	Preemptive strategy	TB reactivation (%)
Current study	2025	Mexico	Yes	auto-HSCT (n = 210)	25.7	INH	1.2
				allo-HSCT (n = 128)	D14.8/R31.3		
Hyun et al.[19]	2024	South Korea	Yes	auto-HSCT (n = 9275)	N.A.	None	0.9
				allo-HSCT (n = 11202)			1.7
Castro-Lima et al.[14]	2023	Brazil	Yes	auto-HSCT (n = 153)	17.1	INH	0
				allo-HSCT (n = 38)			
Zhang et al. [28]	2023	China	Yes	CD-19/CD-22 CAR-T (n = 427)	N.A.	None	1.2
Mert et al.[33]	2022	Turkey	No	auto-HSCT (n = 227)	18.2	INH	0
				allo-HSCT (n = 134)	21.6		
Compagno et al.[34]	2022	Italy	No	auto-HSCT (n = 51)	13.7	INH	0
				allo-HSCT (n = 209)	6.7		
Bello et al.[23]	2022	Philippines	Yes	auto-HSCT (n = 31)	33.3	None	0
				allo-HSCT (n = 2)			
de Oliveira-Rodrigues et al.[13]	2021	Brazil	Yes	allo-HSCT (n = 126)	8.7	INH	0
Anand et al.[30]	2020	USA	No	anti-PD-1/PD-L1 ICI (n = 62823)∞	N.A.	None	0.1
Langan et al.[29]	2020	Germany	No	anti-PD-1/PD-L1 ICI (n = 70)	13.0	INH ± RMP	0
Park et al.[8]	2020	South Korea	Yes	allo-HSCT (n = 1162)	16.0	INH	0
						None	4.6
Zeng et al.[6]	2020	China	Yes	allo-HSCT (n = 6236)	N.A.	INH¢	0.5
Bourlon et al.[12]	2020	Mexico	Yes	auto-HSCT (n = 165)	20.0	INH	0
				allo-HSCT (n = 125)	D41.2/R20.0		
Sosa-Moreno et al.[35]	2020	USA	No	auto-HSCT (n = 217)	5.5	INH	0
				allo-HSCT (n = 240)	1.7		
Cheng et al.[26]	2019	USA	No	auto-HSCT (n = 1252)	1.2	INH	0
				allo-HSCT (n = 1279)			
Aki et al.[36]	2018	Turkey	No	auto-HSCT (n = 287)	31.6	INH	0
				allo-HSCT (n = 271)		None	0.2
Agrawal et al.[20]	2017	India	Yes	allo-HSCT (n = 175)	N.A.	None	2.8
Lee et al.[21]	2017	South Korea	Yes	allo-HSCT (n = 845)	28.1	INH, None	2.1 5.7
Fan et al.[37]	2015	Taiwan	No	auto-HSCT (n = 672)	N.A.	None	1.0
				allo-HSCT (n = 1368)			2.3
Sester et al. [38]	2014	Europe	No	Unspecified (n = 103)	0–5.8	N.A.	0
Moon et al.[22]	2013	South Korea	Yes	auto-HSCT (n = 100)	26.0	None	0.8
				allo-HSCT (n = 114)			

allo, Allogeneic; auto, Autologous; D, Donor; HSCT, Hematopoietic Stem Cell Transplant; ICI, Immune Checkpoint Inhibitors; INH, Isoniazid; N.A., non available; R, Receptor; RMP, Rifampicin.

# According to the 2023 WHO Global Tuberculosis Report.

∞ By adverse drug reactions associated with ICI use.

¢ INH in patients with antecedent ATBI.

Regarding LTBI screening, there is no definitive gold standard, as both QFT and TST detect Mtb but cannot distinguish between active and latent TB infection [11]. Both tests also demonstrate reduced sensitivity in immunosuppressed patients [15]. Several studies recommend using QFT followed by TST to improve diagnostic accuracy in such patients. At the same time, current guidelines suggest performing a second test when the initial result is negative to increase sensitivity, as the benefits of therapy outweigh the potential risks [7]. Patients testing positive on either test should receive treatment. However, data on HSCT recipients is limited. In our study, initial TST positivity was low at 3.1 %, but increased to 28 % after retesting, suggesting the importance of the booster effect in this population. The rates of LTBI detected by TST and QST screening in HSCT recipients were similar (28 % vs 23.1 %, respectively).

Although WHO and CDC recommend chest X-ray screening with chest −X-ray [9,16], in patients undergoing HSCT or with hematologic malignancies, its sensitivity is limited. Up to 20–40 % of active TB cases may present with a normal radiograph, leading to misclassification as latent disease. In contrast, chest CT can detect mediastinal lymphadenopathy or subtle parenchymal changes, findings reported in about one third of patients with radiograph-negative but microbiologically confirmed TB [17,18]. Therefore, in our protocol, chest CT was systematically performed in patients with a positive TST or QFT, to better exclude ATBI.

The prevalence of LTBI in HSCT recipients is not solely determined by local epidemiology. As shown in Table 2, countries not classified as HBCs exhibit a wide range of LTBI prevalence, from 0 % to 31.6 %. In contrast, HBCs show a prevalence ranging from 8.7 % to 41.2 %. Other contributing factors may include variations in healthcare practices, screening protocols, and the immune status of transplant recipients[19]. Additionally, patients with a history of pretransplant ATBI and extensive GvHD face a higher risk of post-transplant TB reactivation, as exemplified by Agrawal et al. and Zeng et al. [6,20].

According to ECIL, the expected rate of ATBI in HSCT recipients is estimated to be 2.2 %–2.7 % in regions with an intermediate to high TB incidence [7]. In our cohort, two patients developed ATBI post-HSCT, with one case being a reactivation of LTBI, resulting in a cumulative incidence of 0.6 %. This rate is similar to reports from HBCs where INH-based therapy is employed (Table 2), showing LTBI reactivation rates ranging from 0 % to 2.1 % [6,8,[12], [13], [14],^21^]. In contrast, centers not using INH therapy report higher reactivation rates, ranging from 0 % to 5.7 % [8,[19], [20], [21], [22], [23]]. For allo-HSCT recipients specifically, the LTBI reactivation rate is higher, at 0–2.5 % in countries using INH therapy [6,8,13,14,21], compared to 1.7–5.7 % in centers without INH therapy [8,15,[19], [20], [21]]. Allo-HSCT recipients carry a higher risk of reactivation, with relative risks ranging from 2.95 to 13.1 times higher [21,24].

Regarding the safety of INH therapy, despite a high rate of successful treatment completion in LTBI patients, hepatotoxicity remained a challenge, leading to therapy discontinuation in 5.7 % of cases. In the literature, INH monotherapy for LTBI is associated with hepatic injury rates with an odds ratio of 1.11 (0.41–3.15) [25].

Alternative regimens, such as shorter-duration therapies like 3HR (three months of daily rifampin (RMP) plus INH), 4R (four months of daily RMP), or 3HP (three months of weekly rifapentine plus INH), may reduce hepatotoxicity and improve adherence while maintaining efficacy [25]. However, shortages and access to RMP pose challenges to its use in LATAM and worldwide, and the risk of drug interactions remains high. Moreover, rifapentine is not available in our country. Additionally, patients with LTBI undergoing HSCT who did not complete INH therapy, adding it post-transplant has shown to be effective [26].

Aside from HSCT recipients, patients receiving T-cell-modifying therapies, such as chimeric antigen receptor (CAR)-T cells, immune checkpoint inhibitors (ICIs), or bispecific T-cell engagers (BiTEs), may also be at a higher risk of TB reactivation. These therapies can lead to persistent hypogammaglobulinemia and prolonged cytopenia, necessitating continuous monitoring and management [27,28].

Moreover, patients receiving CAR-T cell therapy may be at an increased risk for ATBI, as the use of tocilizumab, an anti-interleukin-6 antibody, is associated with a further increase in the risk of TB reactivation [27]. However, reports on TB reactivation are limited for these therapies; a study by Zhang et al. hints at a comparable risk to patients undergoing HSCT, though more evidence is needed, especially in HBCs [28]. In contrast, two reports on ICIs indicate a LTBI prevalence of 13.0 % and a reactivation rate of 0.1 % in low-burden TB regions (see Table 2) [29,30].

Consistent with findings from other studies, the 24-month OS in auto-HSCT (91.4 %) and allo-HSCT (62.6 %) recipients was not impacted by LTBI status [21,31]. Significantly, none of the 93 reported deaths was attributed to ATBI. RFS was also not influenced by LTBI positivity.

The study had several advantages, including a large number of both auto- and allo-HSCT recipients from an HBC in a region with few reports in this setting. It covered a wide range of hematological neoplasms over a long follow-up period (median of 52 months), and showed good tolerability of INH preemptive therapy. However, the study also had limitations, including its retrospective nature and incomplete screening for nine patients, which may have introduced bias in the results. There was also variability in follow-up duration and screening strategies, as the study predominantly included TST (96 %) with only 13 patients undergoing QFT testing. Integrating sequential QFT plus TST into routine screening protocols in HSCT recipients could enhance resource utilization and improve patient outcomes.

## 5 Conclusions

The implementation of a LTBI protocol involving screening with TST or QFT resulted in the diagnosis of LTBI in 27.8 % of HSCT recipients and 14.8 % of donors, similar to other cohorts from LATAM [12,14]. Practical and comprehensive therapy based on INH is associated with low rates of TB reactivation, both within our institution and globally.

These findings emphasize the importance of rigorous LTBI screening and treatment protocols in endemic countries to mitigate the risks associated with TB in HSCT, particularly in allo-HSCT recipients. Further studies are needed to explore the utility of such protocols for other modalities of effector cell and immune-based therapies beyond HSCT.

## 6 Institutional review board statement

The study was conducted by the Declaration of Helsinki and approved by the Institutional Ethics Committee of Instituto Nacional de Cancerología (protocol code No. 2021/121).

## Informed consent statement

Patient consent was waived due to the retrospective nature of the study and the utilization of anonymized clinical data.

## Data availability statement

This study did not generate public archived datasets. However, upon request, the corresponding author can provide access to the anonymized data utilized in the research.

## CRediT authorship contribution statement

**Irma Karen Pellón-Téllez:** Writing – review & editing, Writing – original draft, Project administration, Methodology, Investigation, Data curation, Conceptualization. **Omar Eduardo Fernandez-Vargas:** Conceptualization, Data curation, Formal analysis, Methodology, Visualization, Writing – original draft, Writing – review & editing. **Patricia Cornejo-Juárez:** Writing – review & editing, Validation, Supervision, Methodology, Investigation, Formal analysis. **Alexandra Martin-Onraet:** Writing – review & editing, Writing – original draft, Visualization, Methodology, Investigation, Data curation. **Luis Felipe Rubalcava-Lara:** Writing – original draft, Visualization, Methodology, Formal analysis, Data curation. **Rosa Adriana Alvidrez-González:** Writing – original draft, Methodology, Investigation, Data curation. **Andrés Bonilla Salcedo:** Writing – original draft, Methodology, Investigation, Data curation. **Luis Valero-Saldaña:** Writing – review & editing, Validation, Investigation, Conceptualization. **Brenda Lizeth Acosta-Maldonado:** Writing – review & editing, Visualization, Validation, Methodology, Conceptualization.

## Funding

This research received no external funding.

## Declaration of competing interest

The authors declare that they have no known competing financial interests or personal relationships that could have appeared to influence the work reported in this paper.
